# The influence of water temperature and accelerometer-determined fight intensity on physiological stress and reflex impairment of angled largemouth bass

**DOI:** 10.1093/conphys/cou057

**Published:** 2014-12-08

**Authors:** Jacob W. Brownscombe, Kelsey Marchand, Kathryn Tisshaw, Victoria Fewster, Olivia Groff, Melissa Pichette, Marian Seed, Lee F. G. Gutowsky, Alexander D. M. Wilson, Steven J. Cooke

**Affiliations:** 1 Fish Ecology and Conservation Physiology Laboratory, Department of Biology, Carleton University, 1125 Colonel By Drive, Ottawa, ON, Canada K1S 5B6; 2 Department of Biology, Laurentian University, 935 Ramsey Lake Road, Sudbury, ON, Canada P3E 2C6; 3 Department of Integrative Biology, University of Guelph, 50 Stone Road East, Guelph, ON, Canada N1H 3R6; 4 Department of Biology, Queen's University, 116 Barrie Street, Kingston, ON, Canada K7L 3N6; 5 Department of Biology, University of Ottawa, 30 Marie Curie, Ottawa, ON, Canada K1N 6N5

**Keywords:** Accelerometer, angling, fishing, largemouth bass, reflex impairment, stress physiology

## Abstract

Release of fish captured by recreational anglers is a common practice due to angler conservation ethics or compliance with fisheries regulations. As such, there is a need to understand the factors that influence mortality and sub-lethal impairments to ensure that catch-and-release angling is a sustainable practice. Longer angling times generally contribute to increased stress and mortality in fish such that reducing these times putatively reduces stress and improves survival. However, the relative importance of fight intensity (rather than simply duration) on fish condition is poorly understood. The objective of this research was to examine the effects of fight intensity on physiological stress and reflex impairment of largemouth bass (*Micropterus salmoides*). The largemouth bass were angled using conventional recreational fishing gear in May (water temperature ∼12°C) and June (∼22°C) of 2014 in Lake Opinicon, Ontario, Canada. Fight intensity was quantified using tri-axial accelerometer loggers mounted on the tips of fishing rods. Upon capture, reflex impairment measures were assessed, and fish were held for 1 h prior to blood sampling for measurement of physiological stress (blood glucose and lactate concentrations and pH). Physiological stress values showed a negative trend with fight duration and total fight intensity, but a positive trend with average fight intensity. Water temperature emerged as the most important predictor of the stress response in largemouth bass, while fight duration and intensity were not strong predictors. Reflex impairment was minimal, but higher reflex impairment scores were associated with elevated blood glucose. Overall, the findings of this study suggest that angling for largemouth bass at colder temperatures (<15°C) causes greater physiological stress than at warmer temperatures (>20°C). Based on our findings, we conclude that fight intensity is likely not to be a major driver of physiological stress in this species using typical largemouth bass angling gear, owing to the relatively short fight times (i.e. <2 min).

## Abstract (precis)

We examined the influence of fight intensity on physiological stress and reflex impairment in largemouth bass during angling events using rod-mounted accelerometers across two temperatures (12 and 22°C). Temperature was the strongest predictor of physiological stress response, while fight intensity was not a strong predictor.

## Introduction

Catch-and-release angling is growing in popularity worldwide as a management and conservation strategy (Cowx 2002; Arlinghaus *et al*., 2007). For example, in Canada, release rates among recreational anglers are approaching 60% nationwide (Brownscombe *et al*. 2014). While catch-and-release angling can be an effective conservation strategy, stressors associated with angling (i.e. hooking, fighting, handling and air exposure) can have negative impacts on fish fitness that may lead to population-level effects (reviewed by Cooke and Suski, 2005; Arlinghaus *et al*., 2007). The fate of angled and released fish is primarily determined by angler behaviour (including gear choice) and environmental conditions (Cooke and Sneddon, 2007), so employing certain angling practices can minimize impacts (Pelletier *et al*., 2007). For example, the minimization of angling times and air exposure and the avoidance of angling at extreme temperatures can reduce physiological stress, behavioural impairment and mortality (Cooke and Suski, 2005).

The largemouth bass (*Micropterus salmoides*) is a warm-water fish in the Centrarchidae family, indigenous to eastern North America (Heidinger, 1975; Warren, 2009) and, due to their popularity with recreational anglers, are now widely distributed in warm freshwater systems worldwide (Quinn and Paukert, 2009). Largemouth bass support massive recreational fisheries, including large competitive fishing industries (Schramm *et al*., 1991; Kerr and Kamke, 2003), and given its popularity for sportfishing, release rates are especially high (Quinn, 1996; Brownscombe *et al*., 2014). The potential impacts of angling on this species are well studied (reviewed by Siepker *et al.*, 2007) and can include physiological stress, behavioural impairment (reduced foraging abilities or nest guarding during reproductive period in the spring) and mortality (Philipp *et al*., 1997; Cooke *et al*., 2002; Suski *et al*., 2003; Thompson *et al*., 2008; White *et al*., 2008). Given the ecological, economic and social importance of largemouth bass, understanding and minimizing the impacts of catch-and-release angling on this species is particularly important.

The duration of angling (between hooking and landing when fish resist capture) is often a significant aspect of angling events (e.g. Meka and McCormick, 2004; Thompson *et al*., 2008) because fish typically fight themselves into exhaustion, which involves the depletion of energy reserves and anaerobic respiration (Kieffer, 2000). Indeed, fight duration has been correlated with stress in a number of fish species, including largemouth bass (Gustaveson *et al*., 1991; Kieffer *et al*., 1995; Thorstad *et al*., 2003). For this reason, reducing fight times is generally thought to minimize stress and fitness consequences (Cooke and Suski, 2005). However, no studies have ever reduced the fight duration experimentally in order to confirm its putative value or examined the effects of fight intensity (the amount that the fish resists capture) on fish stress, impairment or mortality. The objective of the present research, therefore, was to examine the influence of fight intensity on physiological stress and reflex impairment in largemouth bass. We predicted that, while physiological stress and reflex impairment would correlate with fight duration, fight intensity would be a stronger predictor of these stress responses. More specifically, we predicted that the stress response would be equal between shorter, more intense fights and longer, less intense fights. To accomplish this, we used fishing rod-mounted tri-axial accelerometer loggers to quantify fight intensity during capture at two temperatures.

## Materials and methods

### Study site and angling experiments

**Figure 1:**
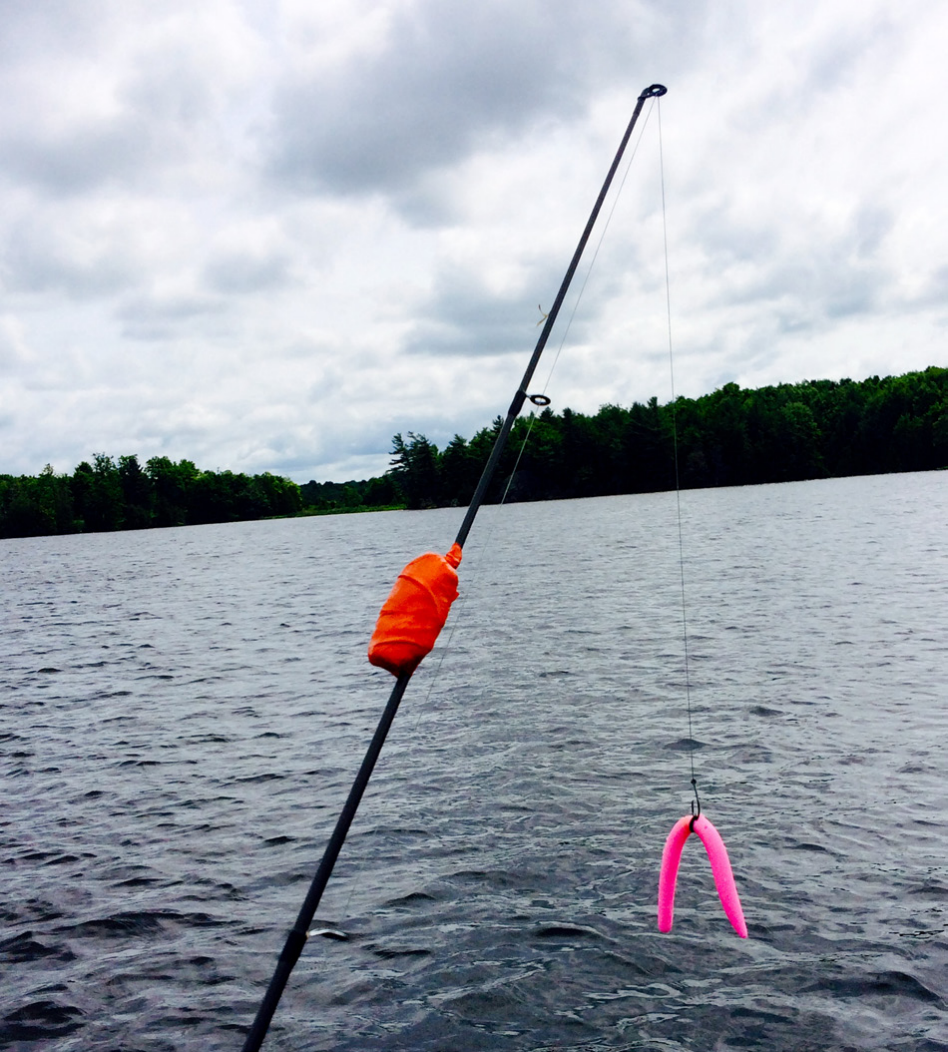
Fishing rod with tri-axial accelerometer attached for measurement of angling fight intensity.

This study was conducted from 1 to 7 May and from 16 to 18 June 2014 in Lake Opinicon, a mesotrophic lake located in eastern Ontario, Canada (44°33′56.0″ N, 76°19′23.6″ W). Angling was conducted using 2-m-long, medium-strength fishing rods and reels equipped with 6.8 kg break-strength, braided Dacron^®^ fishing line, which is typical gear for anglers targeting this species. Terminal tackle included a 1/0 circle hook, baited with a 15 cm wacky-rigged plastic worm that was fished passively. In order to quantify fish fight intensity, tri-axial accelerometer loggers (model X8M, 500 mA h battery, 15 g in air, 25 Hz recording frequency; Gulf Coast Data Concepts) were mounted tightly (with electrical tape) on the fishing rods between the second and third line guides, 20 cm from the tip of the rod (Fig. 1). Accelerometer loggers have been widely used over the past several years to study the energetics and behaviour of free-living animals (for review, see Brown *et al*., 2013). However, the only application in ­recreational fisheries that we are aware of involved attaching accelerometers to fish to assess post-release behaviour (i.e. Brownscombe *et al*., 2013).

When a largemouth bass strike was detected, the angler set the hook into the fish's mouth as per typical angling events and noted the timing of the hook set. Anglers maintained the position of the rod as consistently as possible to isolate the influence of the pull upon the rod by the fish as the main source of acceleration. Upon landing with a rubberized fishing net, the time was noted, and fish were air exposed for a standardized 30 s period, during which time the hook was removed. After the air exposure period, fish were placed in a 90 litre cooler, which was regularly flushed with ambient lake water.

### Reflex indicators

Once placed in the holding containers, fish were assessed for reflex impairment using methods developed under the codification of reflex action mortality predictors (RAMP; Davis, 2010). Five RAMP parameters were assessed, as follows: equilibrium, tail grab, head complex, vestibular–ocular response (VOR) and body flex. Equilibrium was assessed by flipping the fish upside down in water; a positive response was indicated by the fish righting itself within 3 s. Tail grab was measured by grabbing the fish's caudal peduncle while in water; a positive response was indicated by an attempt to escape from the handler. Head complex was assessed by observing the fish's opercula; regular opercular beats indicated a positive response. The VOR was measured by rolling the fish side to side in the longitudinal axis; a positive response was indicated by the fish's eyes moving back and forth, tracking level. Body flex was assessed by lifting the fish into the air by the centre of the body; a positive response was indicated by the flexing or movement of the body. These predictors were used because they are consistent indicators of vitality in teleost fishes (Davis, 2010; Raby *et al*., 2012; Brownscombe *et al.*, 2013, 2014). Each indicator was scored as 1 = impaired and 0 = unimpaired, and overall RAMP scores were calculated as the proportion of indicators impaired.

### Blood physiology

Fish were retained in holding containers for 1 h prior to phlebotomy because physiological stress typically peaks ∼1 h post-stressor in most teleost fishes (Flodmark *et al.*, 2002; Bracewell *et al*., 2003; Jentoft *et al.*, 2005; Suski *et al*., 2007). After the 1 h period, fish were transported to a sloped trough filled with freshwater for drawing of ∼1 ml of blood via caudal venipuncture using an 18-gauge syringe and 3 ml Vacutainer^®^ (lithium heparin). Blood was analysed immediately using point-of-care devices calibrated for fish (see Cooke *et al.*, 2008; Stoot *et al.*, 2014) for blood-plasma lactate (in millimoles per litre; Lactate Pro LT-1710; Akray Inc., Kyoto, Japan), glucose (in millimoles per litre; Accu-Chek Compact Plus; Roche Diagnostics, Basel, Switzerland) and pH (HI-99161; Hanna Instruments, Woonsocket, RI, USA).

### Data analysis

Tri-axial accelerometers recorded total acceleration (*g*) at 25 Hz in three axes (*x*, *y* and *z*), where *g* was the sum of both dynamic (fishing rod) and static (gravity) acceleration, with maximal values of ± 8 *g*. Acceleration sum vectors (SV; *g*) were calculated as SV = √(*x*^2^ + *y*^2^ + *z*^2^) for the duration of each angling event to quantify total and average fight intensities (*g*).

Initially, Pearson's *r* correlations were calculated between angling fight metrics (fight duration, average fight intensity and total fight intensity) and fish metrics (length, blood glucose, blood lactate and blood pH). The above metrics were compared between angling temperatures (12°C in May and 22°C in June) using Welch's *t*-tests. To examine the relationships between RAMP scores and fight metrics, fight duration, total fight intensity and average fight intensity, as well as blood glucose, lactate and pH were compared between unimpaired fish (RAMP = 0) and impaired fish (RAMP ≥ 0.2) with Welch's *t*-tests. To determine significant predictors of physiological stress responses in largemouth bass, linear mixed effects models were generated for blood lactate and glucose concentrations, as well as blood pH. Fixed effects included fight time, mean fight intensity, temperature and the interactions between mean fight intensity × temperature and fight time × mean fight intensity, with fishing rod (angler) as a random effect. Candidate linear models were generated using all combinations of predictors and compared using second-order Akaike information criterion (AICc). The level of significance was set at *P* ≤ 0.05, and all analyses were conducted using RStudio (version 0.97.314). All data are presented as means ± SD (R Core Team, 2013).

## Results

**Figure 2:**
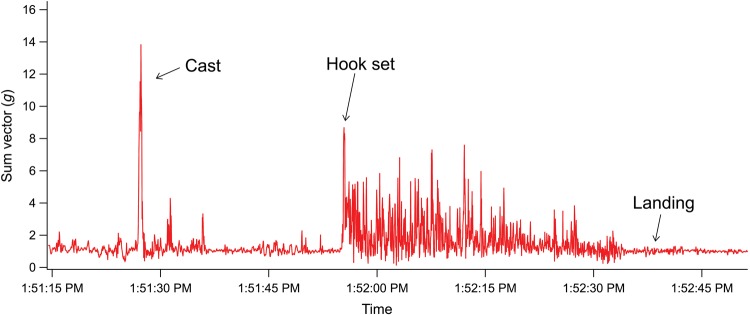
Accelerometric signature (sum vector; *g*) of a largemouth bass angling event.

A total of 86 largemouth bass (356 ± 57 mm total length) were angled at temperatures of 12 ± 0.9°C in May and 22 ± 0.9°C in June. The mean fight duration was 34 ± 13 s (range 9–69 s), while total fight intensity was 1364 ± 463 *g* (range 442–2666 *g*) and average fight intensity was 1.7 ± 0.34 *g* (range 1.2–2.9 *g*), measured using the rod-mounted accelerometers (Table 1 and Fig. 2). Mean blood glucose concentration in angled largemouth bass 1 h after angling events was 6.3 ± 2.5 mmol/l, lactate concentration was 9.6 ± 1.3 mmol/l and pH was 7.4 ± 0.2 (Table 1). Angled largemouth bass exhibited relatively low reflex impairment, with 43% fish exhibiting no impairment (RAMP score = 0), whereas 56% of fish exhibited impaired body flex response (RAMP = 0.2), and one fish also had impaired equilibrium and tail grab responses (RAMP = 0.6). There were no significant differences in fight duration, total fight intensity, average fight intensity, blood pH or lactate between fish that showed reflex impairment and those that were unimpaired (Welch's *t*-test, *t* < 2.0, *P* > 0.05); however, blood glucose was significantly higher in impaired fish than unimpaired fish 1 h after angling events (*t* = 2.6, d.f. = 78.6, *P* = 0.01).

**Table 1:** Angling metrics for largemouth bass

Variable	Mean ± SD (range)
Fish total length (mm)	356 ± 58 (225–486)
Fight duration (s)	33 ± 13 (9–69)
Total fight intensity (*g*)	1364 ± 463 (442–2666)
Average fight intensity (*g*)	1.7 ± 0.3 (1.2–2.9)
Reflex action mortality predictors (RAMP)	0.1 ± 0.1 (0.0–0.6)
Blood glucose (mmol/l)	6.3 ± 2.5 (2.2–13.8)
Blood lactate (mmol/l)	9.6 ± 1.3 (4.3–12.2)
Blood pH	7.4 ± 0.2 (7.0–7.8)

**Figure 3:**
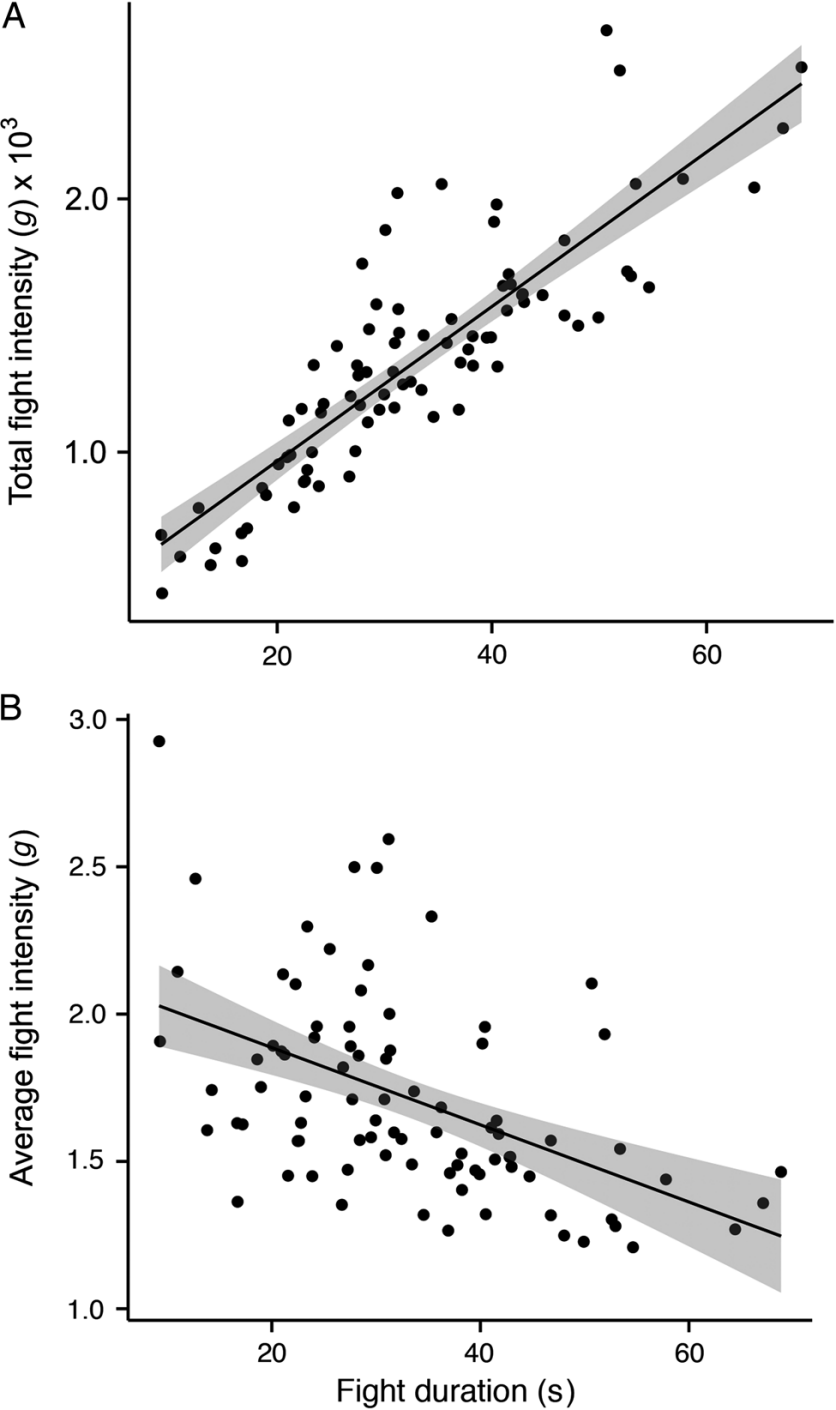
Relationship between fight duration (seconds) and (**A**) total fight intensity (*g*), and (**B**) average fight intensity (*g*) during angling events.

**Figure 4:**
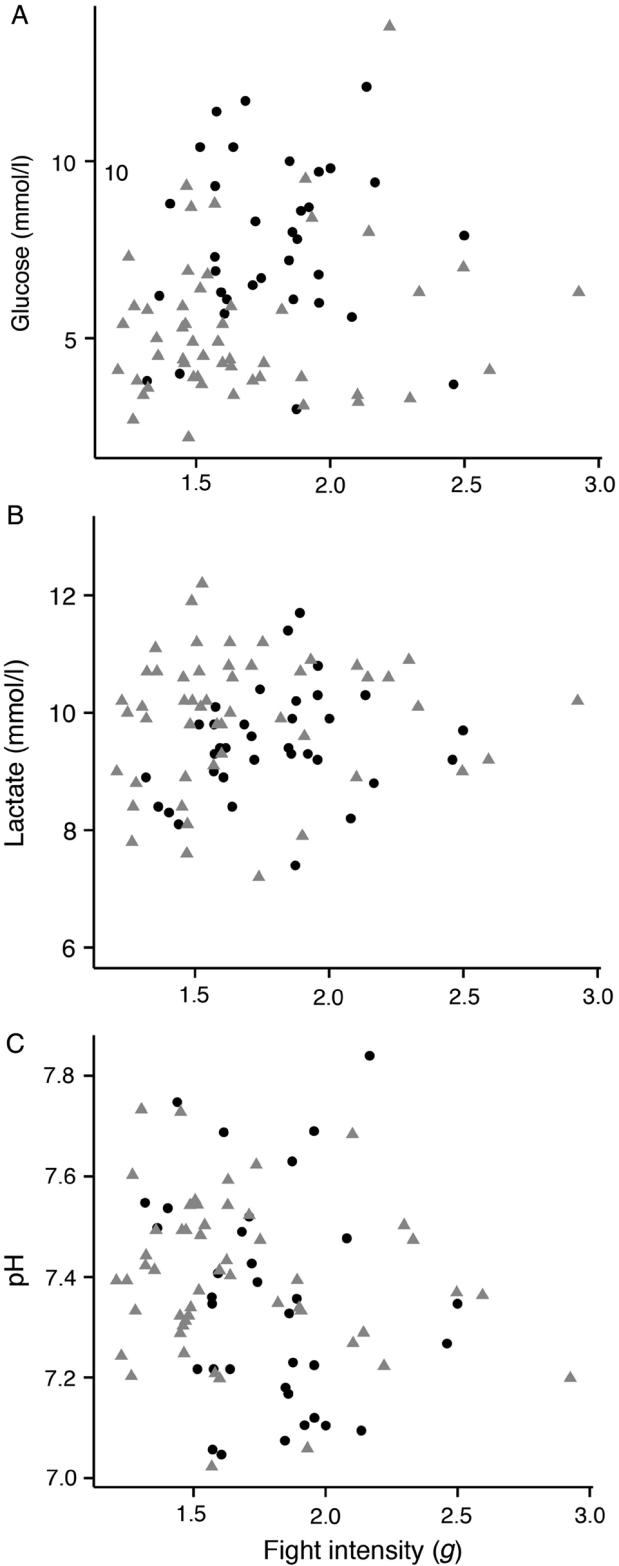
Relationship between average fight intensity and (**A**) blood glucose (mmol/l), (**B**) blood lactate (mmol/l), and (**C**) blood pH at 12°C (black dots) and 22°C (grey triangles) 1 h after angling events.

Fight intensity was significantly correlated with fight duration, where longer fights had higher total acceleration intensities, but lower average intensities (Table 2 and Fig. 3). Fight duration was not correlated with any physiological stress variables, and blood glucose and lactate concentrations even decreased with increasing fight duration, while blood pH increased with increased duration. Fight duration was positively correlated with fish length (Table 2). Blood glucose and lactate increased with average fight intensity and decreased with blood pH; however, only glucose was almost significantly correlated (*P* = 0.057; Table 2 and Fig. 4). Similar to fight duration, fight intensity showed a significant positive correlation with fish length, despite the fact that the two were ­negatively correlated (Table 2). Fish length was also significantly correlated with blood glucose, where larger fish generally had higher glucose concentrations (Table 2). Glucose was significantly higher in fish caught at 12 than at 22°C (Welch's *t*-test; *t* = −4.7, d.f. = 66, *P* < 0.001), but lactate (*t* = −1.0, d.f. = 83.9, *P* = 0.31), pH (*t* = −1.1, d.f. = 54, *P* = 0.26) and fight intensity (*t* = −1.8, d.f. = 83, *P* = 0.07) were not significantly different between temperatures. However, significantly larger fish were caught at 12 than at 22°C (*t* = 3.0, d.f. = 60, *P* = 0.004).

**Table 2:** Pearson's *r* correlation coefficients (bottom left) and corresponding *P*-values (italics; upper right) of largemouth bass angling metrics

	Duration	Average intensity	Total intensity	Length	Glucose	Lactate	pH
Duration		*<0*.001*	*<0*.001*	*0*.005*	*0*.*51*	*0*.*46*	*0*.*09*
Average intensity	−0.49		*0*.*86*	*0*.05*	*0*.*06*	*0*.*23*	*0*.*12*
Total intensity	0.85	−0.02		*<0*.001*	*0*.*67*	*0*.*84*	*0*.*29*
Total length	0.3	0.21	0.45		*0*.04*	*0*.*37*	*0*.*57*
Glucose	−0.07	0.21	0.05	0.22		*0*.*54*	*<0*.001*
Lactate	−0.08	0.13	−0.02	−0.1	0.07		*0*.*49*
pH	0.19	−0.17	0.12	0.06	−0.4	−0.08	

Asterisks indicate significant correlations at 84 degrees of freedom.

The final linear mixed effects model for largemouth bass blood lactate concentrations included mean fight intensity and water temperature; however, neither variable was a significant predictor of blood lactate (Table 3 and Fig. 4). Mean fight intensity and temperature were also included in the final model for blood glucose, but temperature was the only significant predictor. Lastly, fight time was the only predictor in the final model of blood pH, but was not a significant predictor (Table 3 and Fig. 4).

**Table 3:** Final linear mixed effects models of largemouth bass blood glucose and lactate concentrations and blood pH 1 h after angling events

Variable	Factor	Value	SEM	d.f.	*t*-Value	*P*-Value
Lactate	(Intercept)	8.45	0.75	79	11.25	<0.001
	Intensity	0.56	0.40	79	1.40	0.16
	Temperature	0.33	0.28	79	1.17	0.25
Glucose	(Intercept)	6.00	1.30	79	4.60	<0.001
	Intensity	0.92	0.70	79	1.32	0.19
	Temperature	−2.20	0.49	79	−4.48	<0.001
pH	(Intercept)	7.30	0.05	80	138.11	<0.001
	Fight time	0.003	0.001	80	1.74	0.09

## Discussion

It is well established that longer angling, air exposure and handling times can cause greater physiological and ­behavioural impairment in largemouth bass (Gustaveson *et al*., 1991; Thompson *et al*., 2008; White *et al*., 2008). Here, we attempted to isolate and quantify the effects of fighting intensity on physiological stress and reflex impairment in largemouth bass using novel fishing rod-mounted accelerometers at two water temperatures. Interestingly, fight duration and total fight intensity were not correlated with any measured physiological stress responses (blood glucose and lactate concentrations and pH) or reflex impairment scores in angled largemouth bass. However, longer fights had lower average fight intensities, while average fight intensity did correlate positively with blood glucose and lactate concentrations and negatively with blood pH (Table 1). Blood glucose concentrations were significantly higher at colder water temperatures, when average fight intensities were also higher. Furthermore, larger fish had higher average fight intensities, larger fish on average were caught at colder ­temperatures, and blood glucose concentrations were positively correlated with fish size. While it is difficult to isolate the relative contributions of these factors to the blood glucose response of largemouth bass, temperature emerged as the only significant predictor of blood glucose 1 h after angling events, while there were no significant predictors measured here of blood lactate or pH.

While stress responses have been well studied in largemouth bass, no studies have examined their response at temperatures as cold as those included in the present study (mean 12°C during May sampling). In fact, blood glucose concentrations were significantly higher at these colder temperatures than at 22°C. Thompson *et al*. (2008) also found that stress responses, including glucose concentrations, were significantly higher at cooler temperatures (∼15 compared with ∼21°C). This could be because largemouth bass are further from their metabolic optima (see Kieffer and Cooke, 2009) and experience greater anaerobic respiration at lower temperatures (Thompson *et al*., 2008). However, when comparing stress responses at different times of the year, other factors, such as prey availability, gut fullness or reproductive status, may also vary. Indeed, at the lower temperatures in the present study largemouth bass are typically preparing for reproduction and more readily mobilize endogenous energy reserves or have higher gut fullness (Adams *et al*., 1982). In eastern Ontario, where the present study was conducted, targeting largemouth bass during this season is prohibited by law; however, largemouth may be caught frequently while targeting other species with open seasons, such as bluegill sunfish (*Lepomis macrochirus*) or walleye (*Sander vitreus*). Furthermore, in some areas of Canada and the USA, largemouth bass angling is legal during seasons when temperatures are lower. During these time periods, catch-and-release angling of largemouth bass may be inherently more stressful than at warmer temperatures after the spawning period. Cooke and Suski, 2005 called for additional research on the physiological consequences of catch-and-release angling at low water temperatures, which seems merited given our findings.

Angling fights with higher mean intensity did trend towards greater physiological stress responses in largemouth bass, while temperature and mean fight intensity comprised the best model for blood glucose concentrations. However, neither fight intensity nor duration were significant predictors of the stress responses measured here 1 h after landing. While it is intuitive that more intense fighting responses entail greater anaerobic exercise, resulting in a greater stress response, our findings did not strongly support this prediction. It is also surprising that blood lactate and pH, which are also typically reflective of anaerobic exercise, were not related to any fight metrics or environmental variables measured here. White *et al*. (2008) found that exercising largemouth bass for 180 s caused significantly higher blood lactate concentrations and haematocrit than at 20 s, but found no significant differences in blood glucose concentrations. Meka and McCormick (2004) also found that rainbow trout (*Oncorhynchus mykiss*) with longer fight times (>2 min) had significantly higher blood lactate and cortisol concentrations; however, larger fish also had longer fight times. Indeed, it is difficult to disentangle the effects of fish size and fighting metrics on fish stress response using ‘natural’ angling events because these metrics are so commonly correlated. Perhaps, if we had collected blood samples at a different time (e.g. 30 min post-stressor, while peaking, or 2 h post-stressor, when recovery would typically be underway), we might have detected such a relationship. The results of the present study may simply reflect the fact that fight duration and intensity are not major factors contributing to the stress response of largemouth bass with typical gear and conditions (all fight times in the present study were <2 min with 6.8 kg break-strength line and medium-strength rods).

Greater durations of air exposure have shown to increase stress responses (Cooke *et al*., 2002; Thompson *et al.*, 2008), and collectively, research on largemouth bass suggests that using typical bass fishing gear and minimizing or eliminating air exposure results in relatively low stress, few reflex impairments and high survival (reviewed by Siepker *et al.*, 2007). Here, we used a standardized duration of air exposure (30 s) to examine the relationship between fight intensity, stress and reflex impairment. There may also be an interaction between fight intensity while on the line and subsequent air exposure duration in real angling scenarios. For example, fish that fight more vigorously may be more exhausted and less active during handling, reducing air exposure times. Future studies should aim to examine how various aspects of angling practices interact and influence fish condition upon release.

The ability to assess fish condition prior to release has important applications for fish conservation. For example, anglers can make educated decisions about whether to release a fish immediately, retain it for a short recovery period or harvest it for consumption. It may also serve as a practical proxy for fish survival for fisheries managers to incorporate post-release mortality into management strategies. In the present study, all largemouth bass exhibited relatively low reflex impairment with limited (30 s) amounts of air ­exposure and <2 min fight times. However, over 50% of fish lacked a body flex response when removed from water, and these fish had significantly higher blood glucose concentrations 1 h post-angling than fish showing no impairment. Reflex impairment measures, e.g. RAMP (Davis, 2010), are consistent indicators of behavioural impairment and mortality across fish species (e.g. Davis and Ottmar, 2006; Davis, 2007; Raby *et al*., 2012; Brownscombe *et al.*, 2013); however, the connection of RAMP with physiological stress is more tenuous. Here we found that impaired fish did have significantly higher blood glucose, but there was no relationship with RAMP and any other physiological stress measure. Future work should aim to test the applicability of reflex tests further in predicting stress, behavioural impairment and mortality for this species to contribute to its conservation.

In conclusion, angling for largemouth bass at cold temperatures prior to the spawning period caused greater physiological disturbance in blood glucose concentrations than at warmer temperatures, which may be related to temperature and/or seasonal changes in the physiological status of the fish prior to angling. Our findings suggest that the fight, where fish resist capture prior to landing, has limited influence on physiological and behavioural impairment of largemouth bass when fight times are relatively low, using typical largemouth bass angling gear. Reflex impairment measures reflect underlying physiological stress in this species and may have further applications for assessing the condition of largemouth bass prior to release. Future research should aim to understand how fight duration and intensity mechanistically contribute to fish stress in species that typically exhibit greater fight times (e.g. rainbow trout, bonefish, *Albula* spp*.* or billfish) to determine whether minimization of fight times is effective at reducing physiological stress and negative fitness consequences.

## Funding

This work was supported by the Natural Sciences and Engineering Research Council of Canada. The research vessel used for this research was funded by the Canada Foundation for Innovation and the Ontario Research Fund.
